# Tunable plasmons in regular planar arrays of graphene nanoribbons with armchair and zigzag-shaped edges

**DOI:** 10.3762/bjnano.8.18

**Published:** 2017-01-17

**Authors:** Cristian Vacacela Gomez, Michele Pisarra, Mario Gravina, Antonello Sindona

**Affiliations:** 1 Dipartimento di Fisica, Università della Calabria, Via P. Bucci, Cubo 30C, 87036 Rende (CS), Italy; 2 INFN, sezione LNF, Gruppo collegato di Cosenza, Via P. Bucci, Cubo 31C, 87036 Rende (CS), Italy; 3 Departamento de Química, Universidad Autónoma de Madrid, Calle Francisco Tomás y Valiente 7 (Módulo 13), 28049, Madrid, Spain

**Keywords:** graphene nanoribbons, plasmonics, time-dependent density functional theory

## Abstract

Recent experimental evidence for and the theoretical confirmation of tunable edge plasmons and surface plasmons in graphene nanoribbons have opened up new opportunities to scrutinize the main geometric and conformation factors, which can be used to modulate these collective modes in the infrared-to-terahertz frequency band. Here, we show how the extrinsic plasmon structure of regular planar arrays of graphene nanoribbons, with perfectly symmetric edges, is influenced by the width, chirality and unit-cell length of each ribbon, as well as the in-plane vacuum distance between two contiguous ribbons. Our predictions, based on time-dependent density functional theory, in the random phase approximation, are expected to be of immediate help for measurements of plasmonic features in nanoscale architectures of nanoribbon devices.

## Introduction

Quantized, coherent and collective density fluctuations of the valence electrons in low-dimensional nanostructures, better known as plasmons, have been attracting significant interest, due their capability to couple with light and other charged particles, thus paving the way to novel applications in a wide range of technologies, as diverse as biosensing, light harvesting or quantum information [1–5]. On more fundamental grounds, plasmon-like modes are the “true” low-energy excitations of low-dimensional systems [6–7], while charged and spinful modes are realized as coherent states, with their own peculiar dynamics [8–9], both in normal superconducting phases [10–14]. Graphene has first emerged as an extraordinary platform for controlling the propagation of surface-plasmon waves [15], because of its unique electronic and optical properties [16]. In particular, the extrinsic plasmons of this one-atom-thick hexagonal lattice of sp-bonded carbon atoms have shown much stronger confinement, larger tunablity and lower losses with respect to more conventional plasmonic nanoparticles, such as, for example, silver and gold [17]. With the rise of low-dimensional materials, a number of theoretical and experimental studies have been oriented to launch, control, manipulate and detect plasmons in graphene-related and beyond-graphene structures [18–21], which are expected to be embedded in next-generation nano-devices that may operate from infrared (IR) to terahertz (THz) frequencies [22–25]. As a noteworthy example, graphene nanoribbons (GNRs) preserve most of the exceptional features of graphene, with the additional property that they are semiconductors and their band gap is geometrically controllable.

A clear picture of confined edge (interband) and surface (intraband) plasmons in GNRs, as wide as 100–500 nm, has been achieved by infrared imaging measurements on the nanoscale [26]. On the theoretical side, some nearest-neighbor tight-binding [27–28] and semiclassical electromagnetic [29–30] approaches have been able to characterize the intraband mode, being generally excited by a THz electromagnetic field pulse. Very recently, an ab initio analysis has elucidated the role of both intraband and interband plasmons in narrow GNRs below ca. 1 nm in width [31].

In this work, we extend the results of [31], to include some GNRs up to 2.2 nm wide, and demonstrate how the extrinsic plasmons of these systems can be finely tuned by changing a small number of simple parameters, such as the unit-cell length, width and chirality of each GNR, or the in-plane distance between contiguous GNRs. Our study is carried out using time-dependent (TD) density functional theory (DFT), within the random phase approximation (RPA). The computations are performed at room temperature (*T* = 300 K), including both intrinsic and extrinsic conditions. Different zigzag (Z) and armchair (A) configurations are examined, with the GNR-ends being passivated by hydrogen atoms, which mimics an ideal setup of long ribbons, with perfectly symmetric edges, suspended or grown on inert substrates. A specific focus is made on the 5AGNR, 11AGNR and 4ZGNR, 10ZGNR geometries that are, respectively, characterized by 4,10 zigzag-chains and 5,11 dimer-lines across the width of the GNRs. The dielectric properties of these systems are calculated in response to probe electrons or photons with incident energies, ω, smaller than 20 eV, and in-plane incident momentum **q** below 0.8 Å^−1^. For comparison purposes, the well-known intrinsic plasmonics of graphene are also reported.

In the following, we briefly account for the theoretical tools that we have used to explore the electronic structure and dielectric properties of 5AGNR, 11AGNR and 4ZGNR, 10ZGNR and graphene (Results and Discussion, chapter 'Theoretical framework'). Next, we will present a detailed analysis of the changes induced by the above mentioned parameters to the peak position, intensity, and dispersion of the GNR-plasmons (Results and Discussion, chapter 'Tunable plasmons in GNR arrays').

## Results and Discussion

### Theoretical framework

Our TDDFT approach is divided into two steps. First, the (ground-state) electronic properties of the different GNRs (and graphene) are obtained by DFT. Second, the basic equation of linear-response theory in the RPA is employed, with a corrected electron–electron interaction, to calculate the dielectric properties, and hence the plasmon structure, of the systems.

#### DFT method

Density-functional calculations are performed using the plane-wave (PW) basis-set [32], i.e., the normalized space functions 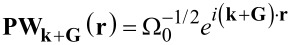, which depend on the wave-vectors **k** of the first Brillouin zone (1st BZ), and the reciprocal lattice vectors **G** associated to the three-dimensional (3D) crystal of unit-cell volume Ω_0_. The ground-state electronic properties of the different GNRs (as well as graphene) are computed within the local density approximation (LDA), being defined by the Perdew–Zunger parameterization of the uniform-gas correlation energy [33]. Norm-conserving pseudopotentials of the Troullier–Martins type are adopted to eliminate the core electrons [34]. A cut-off energy of ca. 680 eV on the number of PWs is sufficient to obtain well-converged electronic structures with ca. 10^5^ PWs.

**Figure 1:**
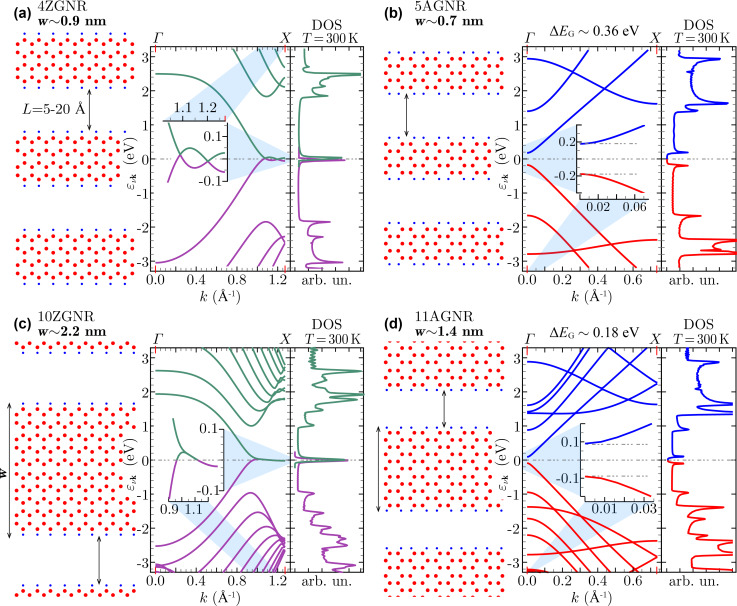
Geometry, LDA band-structure and DOS of the different (zigzag and armchair) GNR arrays reported in the present study, namely 4ZGNR (a), 5AGNR (b), 10ZGNR (c), and 11AGNR (d). In the LDA, the ZGNRs are gapless, while the AGNRs have a band gap Δ*E*_G_, which decreases with increasing the ribbon width *w*, as attested by the insets in the band plots. The ground-state features of the systems are strongly sensitive to the value of the C–C bond-length (here fixed to 1.42 Å) and the ribbon width, while they are practically unaffected by in-plane vacuum distances *L* (here fixed to 15 Å). Figure 1a and Figure 1b are adapted from Figure 1 of [31].

In the GNR arrays, the C–C length is allowed to range from 1.414 to 1.426 Å, while the C–H bond length is fixed to 1.09 Å, with a bond-angle of 120 °(as shown in [31] relaxation effects play a minor role in 4ZGNR and 5AGNR). The 3D periodicity required by PW-DFT is generated by using an in-plane vacuum distance of 5–20 Å, and an out-of-plane lattice constant of 15 Å. The self-consistent run is carried out using an unshifted (and Γ-centered) Monkhorst–Pack (MP) grid, made of 60 × 60 × 1 **k**-points [35], which results in a uniform sampling of the irreducible 1st BZ on the ΓX-segment (shown in Figure 1). The converged electron densities are subsequently used to compute the Kohn–Sham (KS) electronic structure on a denser MP-mesh of 180 × 1 × 1 **k**-points, including up to 120 bands, which is enough to explore the dielectric properties of the GNRs at probing energies below ≈20 eV. The IR to THz region is further scrutinized with a finer MP mesh of 2000 × 1 × 1 **k**-points, including up to 30 bands.

The main results of our DFT computations are summarized in the plots of Figure 1, which show the different geometry, band structure and density of states (DOS) of the GNR arrays. 4ZGNR and 10ZGNR behave as semimetals, with barely touching valence and conduction bands (Figure 1a,c). 5AGNR and 11AGNR are small-gap semiconductors (Figure 1b,d), contrary to nearest-neighbor TB approaches in which all AGNRs appear gapless [27].

Indeed, several DFT studies have carefully characterized the band gaps of ZGNRs and AGNRs [36–39]. In particular, local spin-density calculations suggest the opening of a band gap larger than 0.1 eV in ZGNRs [36–37]. However, the LDA analysis of a virtually gapless GNR, i.e., 4ZGNR or 10ZGNR, in comparison with 5AGNR and 11AGNR, is particularly instructive to emphasize the different role played by doping in separating the extrinsic plasmon modes, which will be detailed in chapter 'Tunable plasmons in GNR arrays'.

The simulations at hand explore ranges of different geometrical and conformation parameters, which will be used to characterize the tunability of the GNR plasmons. In particular, (i) GNR widths (*w*) of around 0.7–2.2 nm are sorted out; (ii) zigzag and armchair edges are considered, to elucidate the role played by chirality; (iii) in-plane vacuum distances from 5 to 20 Å are tested; and (iv) different unit-cell extensions are simulated by changing the C–C bond length by about 0.5%, to account for stretching effects. As for intrinsic graphene, the C–C bond length and out-of-plane lattice constant are fixed to 1.42 Å and 15 Å, respectively. The self-consistent run is performed on a 60 × 60 × 1 MP-grid, and the KS electronic properties are then computed on a 180 × 180 × 1 MP-grid, including up to 80 bands.

#### TDDFT approach

The KS eigenvalues 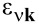 (see Figure 1) and corresponding eigenfunctions 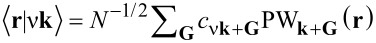 are the main outputs of the DFT computations, with *N* being the total number of **k**-points in the 1st BZ, and ν being the band index. The KS eigensystem 
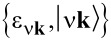
 gives access to the unperturbed density–density response function, of the non-interacting valence electrons, to a probe particle of energy ω and momentum **q**. The latter is provided by the Alder–Wiser formula [40–41]:

[1]
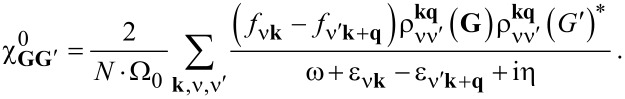


Indeed, plasmons in solid-state materials are typically triggered by electron-beam radiation, photo-currents, and even charged ions, with incident kinetic energies of the order of 0.1–1 keV [42–43]. In the present context, the probe particle is an electron or a photon that weakly perturbs the system.

In Equation 1 (expressed in Hartree atomic units), the factor of two takes into account the electron spin, η indicates an infinitesimal (positive) broadening parameter (set to 0.02 eV), *f*_ν_**_k_** is the Fermi–Dirac distribution, and

[2]
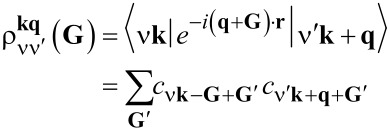


labels the density–density correlation matrix elements, which depend on the number of PWs included in the DFT simulations.

The full susceptibility or interacting density–density response function is determined by the central equation of linear-response TDDFT [44–45]

[3]
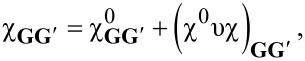


where, in the RPA, the υ**_GG′_** terms are approximated to the bare Coulomb coefficients:

[4]
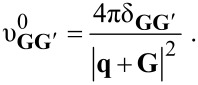


However, the long-range character of the Coulomb potential yields non-negligible interactions between replicas along out-of-plane direction *z*. To erase this unphysical phenomenon, a two-dimensional (2D) cut-off, say, a truncated Coulomb potential is used [46–49] to replace the υ**_GG′_** terms by the truncated Fourier integral:

[5]
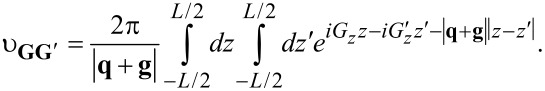


Here, **g** and *G_z_* denote the in-plane and out-of-plane components of **G**, respectively.

With this 2D correction in mind, we can introduce the inverse dielectric matrix:

[6]
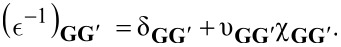


The zeros in the real part of the macroscopic dielectric function (permittivity) provide the condition for a plasmon resonance to occur, stated as:

[7]
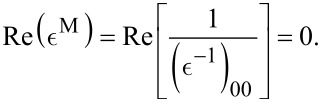


The imaginary part of the inverse permittivity is proportional to so-called energy loss (EL) function, which provides the plasmon structure:

[8]
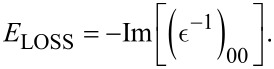


Non-local field effects [50] are included in *E*_LOSS_ through Equation 3. We have verified that ca. 120 **G** vectors for all GNRs (and ca. 51 **G** vectors for graphene), sorted in length and being of the form (0,0,*G_z_*), give well-resolved and converged results in the sampled energy–momentum region, delimited by ω < 20 eV and *q* < 0.8 Å^−1^.

### Tunable plasmons in GNR arrays

**Figure 2:**
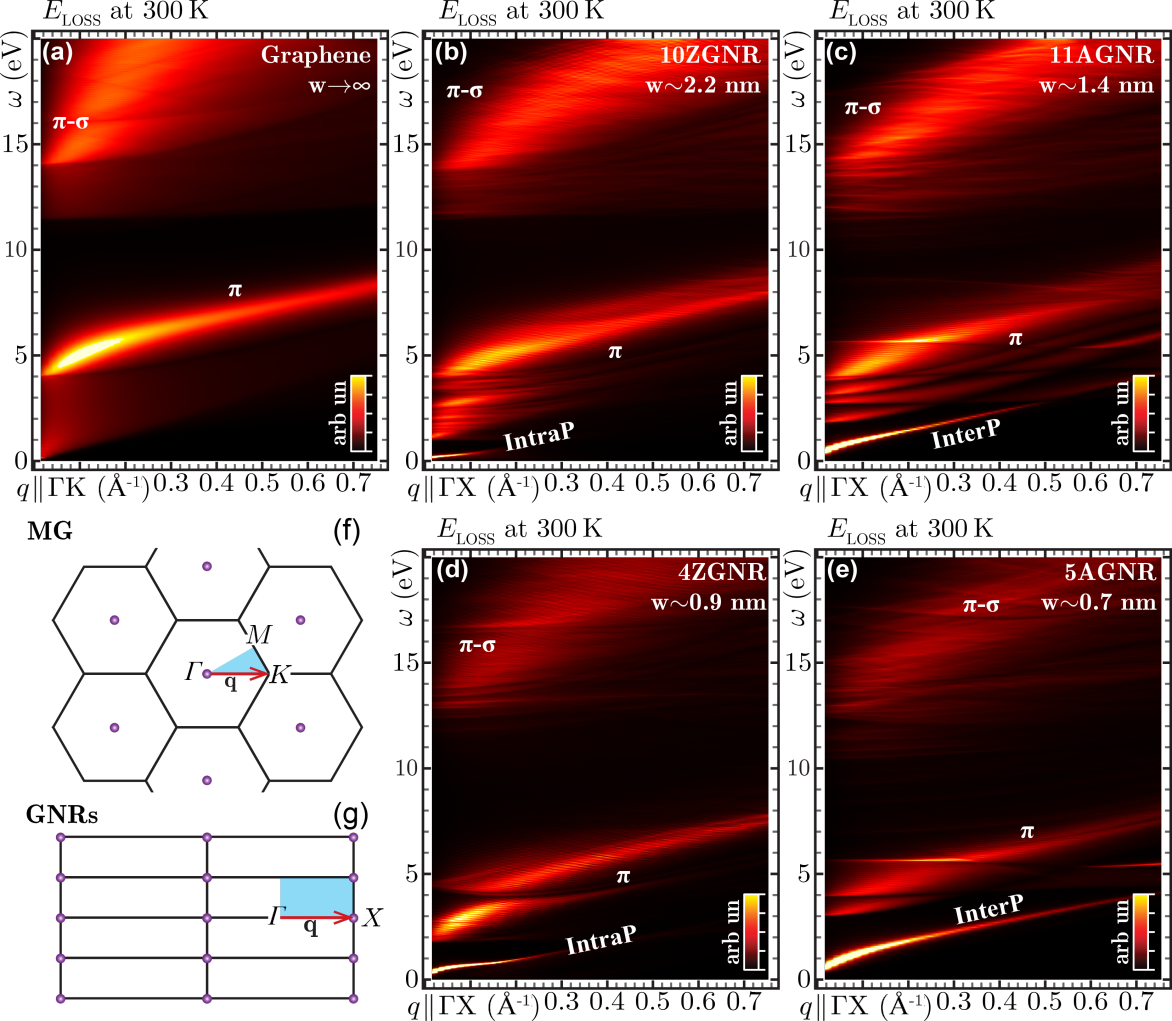
Loss properties of intrinsic graphene (a), i.e., an example of infinite-width GNR, and the undoped GNR arrays of Figure 1 (b–e), computed at room temperature from the TDDFT-RPA machinery summarized by Equations 1–8 of the main text. The EL function of Equation 8 is represented as a density-color plot vs incident energy ω (in eV) and momentum *q* (in Å^−1^). The latter is sampled along the ΓΚ path of the 1st BZ of graphene (f), and the ΓΧ path of the 1st BZ of the different GNRs (g). Besides the π and π–σ plasmons, the ZGNRs present a low-energy intraband mode (IntraP), whereas the AGNRs have a low-energy interband mode (InterP). The intensity scale in (a–d) is cut at 80% of the π peak maximum, to ease comparison between the different density-color plots.

We proceed by clarifying the role played by the geometric and conformation parameters introduced above in the different GNRs, whose intrinsic response is shown in Figure 2 together with that of graphene. In the following, we will also evaluate several extrinsic conditions associated to Fermi energy shifts Δ*E*_F_ in the range of −0.2 to 0.2 eV.

#### Ribbon width and chirality

Independently on the width and chirality of the ribbons, all GNRs are characterized by two interband plasmons at excitation energies above about 2 eV. These excitations, shown in Figure 2b–e, are analogous to the well-known π and π–σ plasmons of graphene [51–52], as displayed in Figure 2a. Similar features occur in bilayer graphene [51], multilayer graphene [52], graphene–metal interfaces [53–59] and graphite [52]. In our calculations, the intensity of the π and π–σ modes increases with increasing the GNR width, reaching its maximum brightness in graphene, which may be seen as a GNR of infinite width.

The energy window displayed in Figure 2 does not show the complete energy–momentum dispersion of the π–σ plasmon, however, the latter seems to be quadratic in graphene and linear in the GNRs. At long wavelengths, in the *q*→0 region, the π plasmon of all systems has a 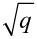-like dispersion, while at *q* > 0.2 Å^−1^ it exhibits linear behavior. The intrinsic plasmons of 10ZGNR and 11AGNR appear in the same energy region as those of graphene, i.e., at ω ≈ 4–5 eV and ω ≈ 14–15 eV. On the other hand, they are red-shifted in 4ZGNR and 5AGNR, with the π plasmon having a peak at ω ≈ 2–3 eV and the π–σ plasmon lying at ω ≈ 13–14 eV. Furthermore, the π and π–σ plasmons detected in 4ZGNRs (w ≈ 0.9 nm) and 5AGNR (w ≈ 0.7 nm) exhibit markedly discontinuous dispersions, being split into more branches [31]. This is a consequence of the narrow widths of the two systems that generate several, distinct one-dimensional bands of π- and σ-character (Figure 1c,d).

By increasing the GNR width (w > 1 nm), the number of bands increases, and less disjoint plasmon dispersions appear, which clearly tend to the continuous patterns of graphene (*w*→∞). Thus, semiconducting and semimetallic GNRs have plasmon resonances in the visible (VIS) to ultraviolet (UV) regime that may be controlled by the GNR width. This tunability feature is evidently absent in graphene.

Quantum confinement and chirality are key factors for plasmon resonances at frequencies smaller than 2 eV. We see that zigzag systems exhibit an intraband plasmon, while armchair systems present an interband plasmon. These two modes correspond to the surface and edge plasmons detected in large-width, extrinsic GNR arrays fabricated on Al_2_O_3_ [26]. The surface plasmon of ZGNRs is originated by the large DOS peak observed at the Fermi level *E*_F_ (Figure 1a,c). This mode shows a 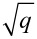-like dispersion [31] and seems to be analogous to the conventional 2D plasmons of extrinsic graphene [19,51]. The edge plasmon of AGNRs appears as an effect of collective excitations generated close to *E*_F_ [31], associated to single-particle excitations that connect the two DOS peaks around *E*_F_ (Figure 1b,d). The characteristics of this interband mode are similar to those of the π plasmon of intrinsic graphene, i.e., at long wavelengths the interband plasmon shows a 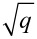-like dispersion, while at *q* > 0.1 Å^−1^ it displays a linear dispersion.

Both the intraband and interband modes have been proved to be genuine plasmons in intrinsic 4ZGNR and 5AGNR [31], as they are strictly associated to the zeroes of the real permittivity, satisfying the condition given by Equation 7. It has been further demonstrated that extrinsic 4ZGNR presents only an intraband plasmon structure, independently on the positive doping level used (below ca. 1 eV), while both intraband and interband plasmons coexist in 5AGNR [31].

**Figure 3:**
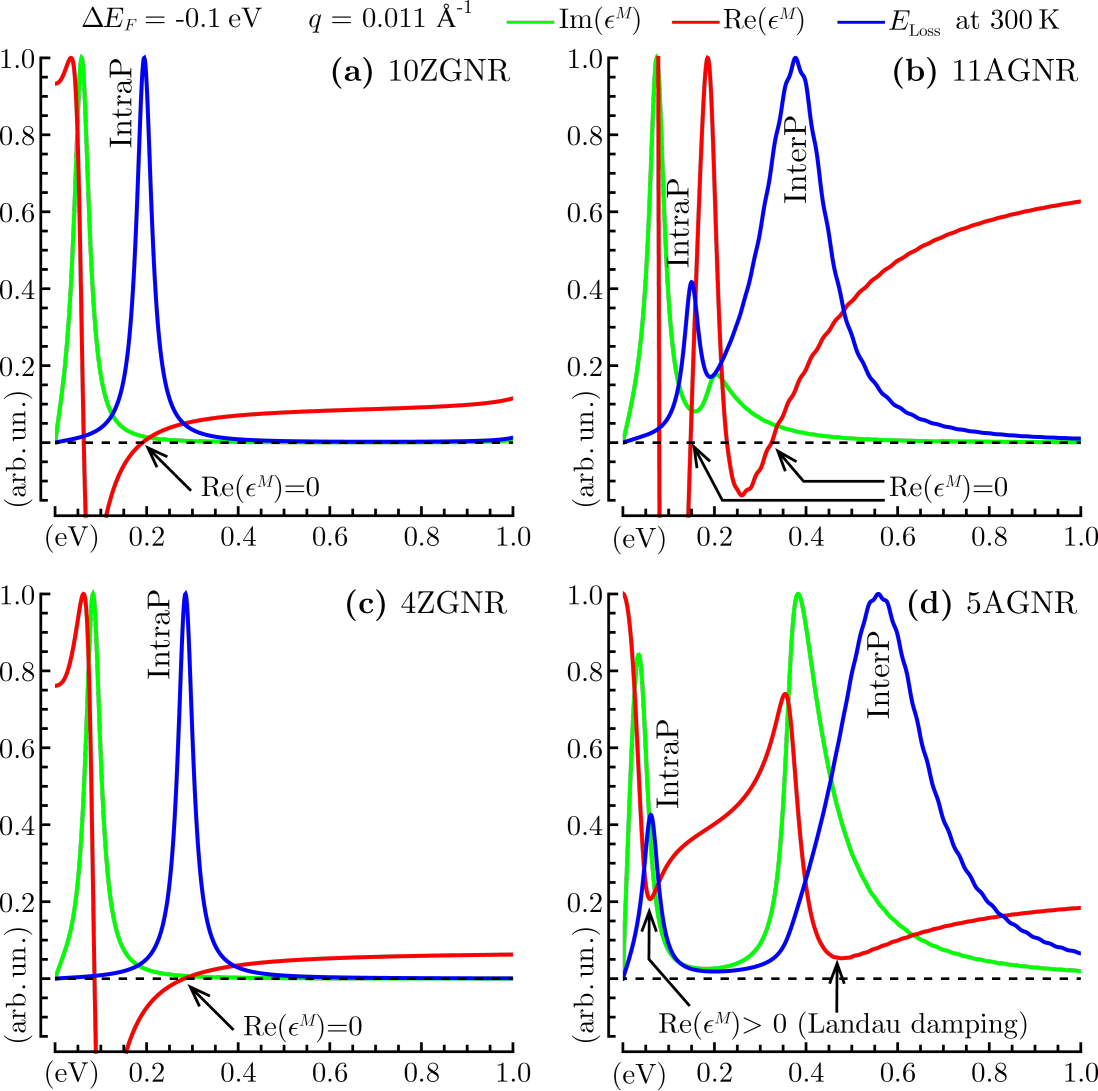
Macroscopic permittivity (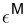, Equation 6) and EL function (*E*_LOSS_, Equation 8) at room temperature for the GNR arrays of Figure 1 and Figure 2, i.e., 10ZGNR (a), 11AGNR (b), 4ZGNR (c) and 5AGNR (d). The energy region ω ≤ 1 eV is explored at a fixed incident momentum *q* = 0.011 Å^−1^ parallel to ΓX (Figure 2g), with a negative doping level Δ*E*_F_ of −0.1 eV. The black arrows mark the positions where the real permittivity has a zero value (a–c) or a minimum (d), which reflects a Landau damping mechanism due to single-particle excitation processes. The intraband and interband plasmons are denoted IntraP and InterP, as in Figure 2.

To support this result, we report in Figure 3 the macroscopic dielectric function and the EL function of the different GNR arrays for a selected momentum value (*q* = 0.011 Å^−1^) and a negative doping level (Δ*E*_F_ = −0.1 eV). We see that 10ZGNR and 4ZGNR present similar plasmonic features, with the intraband plasmon resonance being blue-shifted by increasing the GNR width (Figure 3a,c). In 11AGNR and 5AGNR, not only the peak position but also the interplay of the interband and intraband plasmon is strongly dictated by the doping level and the GNR width (Figure 3b,d).

In 5AGNR, the two modes are well resolved in energy, with the zeroes of the real permittivity being hidden by the Landau damping mechanism, associated to single-particle excitation processes [25,46–48]. In 11AGNR the same modes strongly interfere and largely dominate with respect to single-particle excitations. A similar interplay was observed in extrinsic 5AGNR subject to a positive doping of about 0.3 eV [31]. These outcomes are basically due to the different band-gap values of the two AGNRs, which according to our predictions are ca. 0.18 eV for 11AGNR, and ca. 0.36 eV for 5AGNR. Accordingly, less energy requirements are needed to produce a well-defined intraband collective electronic excitation in 11AGNR. On the other hand, a positive doping larger than 0.2 eV yields a well-defined intraband plasmon in 5AGNR [31]. Interestingly enough, some GNRs with band-gap values of the same order of 11AGNR and 5AGNR have been recently synthetized on Au(111) [60]. Then, our ab initio analysis can be of help in interpreting plasmon measurements on currently synthetized GNR-structures.

Chirality seems to be a major point for the design of GNR-based plasmonic devices. One or two plasmon modes can be exploited, depending on the shape of the GNR edges. In this respect, negative or positive doping acts as a modulating factor of the plasmon modes.

**Figure 4:**
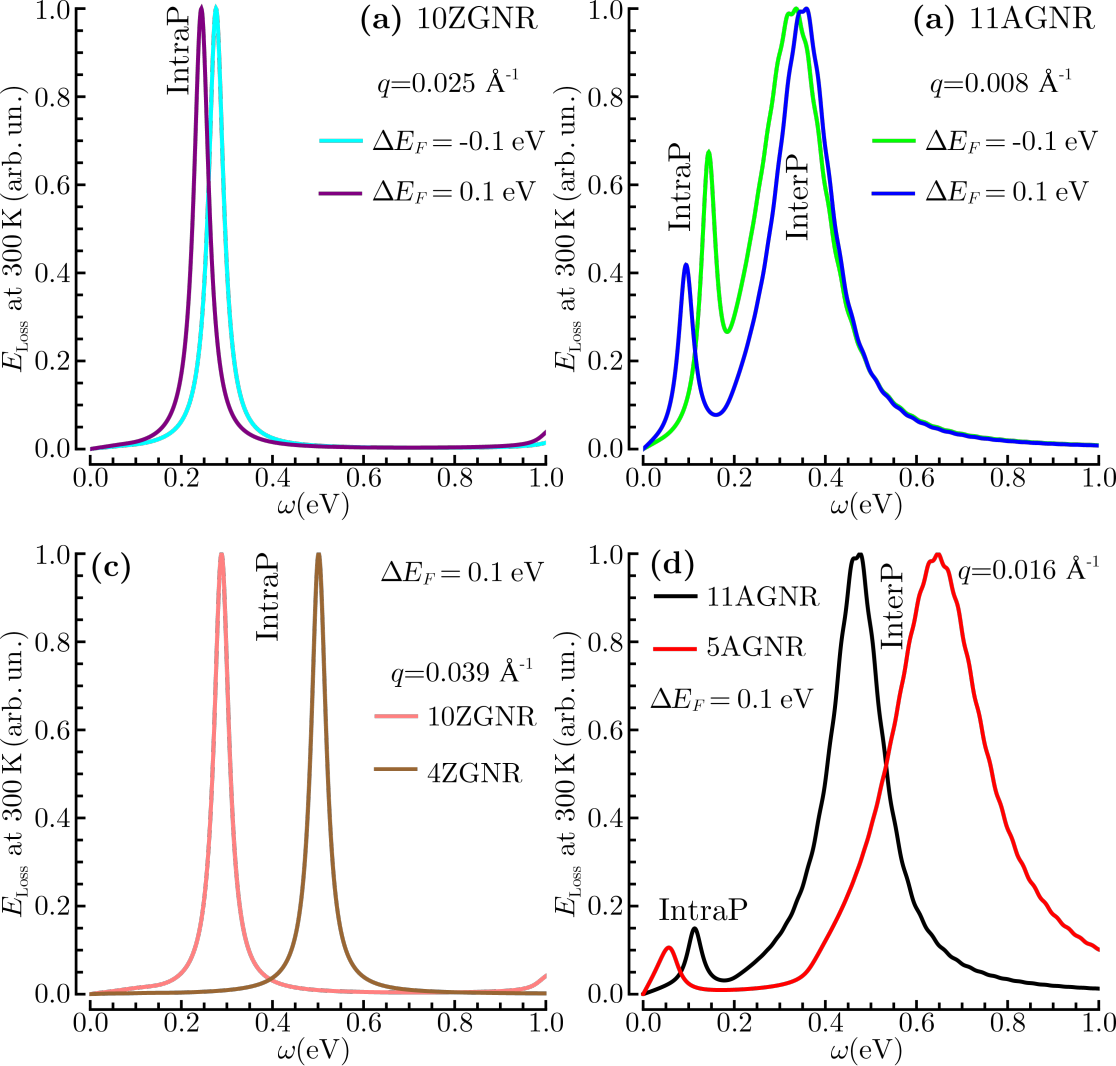
EL function of Equation 8 at room temperature for the GNR-arrays considered in the main text, e.g., 10ZGNR (a), 11AGNR (b), 4ZGNR (c) and 5AGNR (d), being subject to doping levels Δ*E*_F_ of ±0.1 eV. *E*_LOSS_ is plotted in the energy range ω ≤ 1 eV at some fixed transferred momentum values, *q* = 0.025 Å^-1^ (a), 0.008 Å^-1^ (b), 0.039 Å^-1^ (c), 0.016 Å^-1^ (d), parallel to ΓX (Figure 2g). The intraband and interband plasmons are denoted as IntraP and InterP, as in Figure 2 and Figure 3.

In Figure 4 we see that a change in doping sign, from −0.1 to 0.1 eV, produces a slight red shift in the intraband plasmon of 10ZGNR and the interband plasmon of 11AGNR (Figure 4a,b). More significant variations are observed in the intraband plasmon of 11AGNR, which is markedly blue-shifted and doubled in intensity by the same change of extrinsic conditions (Figure 4b). Therefore, an asymmetric response is observed in the intraband plasmon of semiconducting GNRs (Figure 4b). Moreover, as the GNR width decreases an appreciable blue/red shift is detected in the plasmon peaks of both ZGNRs and AGNRs (Figure 4c,d). Thus, a tunable energy response may be more strongly influenced by the ribbon width than the doping level.

#### Mechanical deformations

**Figure 5:**
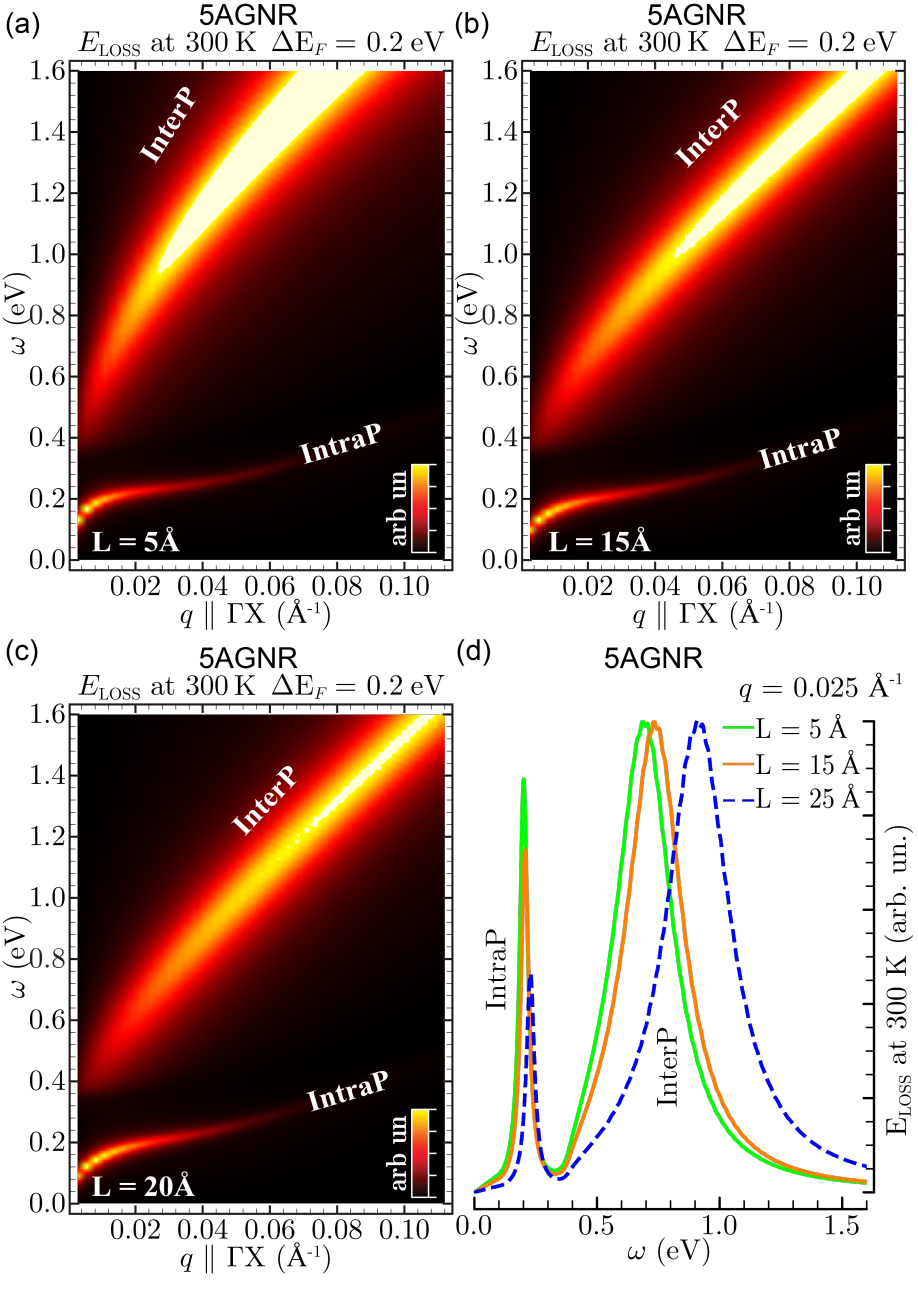
EL function of three positively doped 5AGNRs (Δ*E*_F_ = 0.2 eV) separated by an in-plane vacuum distance *L* of 5 Å (a), 15 Å (b), and 20 Å (c). In (a–c) the energy–momentum region ω ≤ 1.6 eV and *q* || ΓX ≤ 0.1 Å^−1^ is explored, showing how the parameter *L* modifies the relative position between the intraband and interband plasmons, denoted IntraP and InterP as in Figures 2–4. The intensity scale is cut at 95% of the IntraP peak. The effect of changing the in-plane vacuum distance is even more evident in (d), where the different EL functions of (a–c) are compared at a fixed momentum value of *q* = 0.025 Å^−1^ parallel to ΓΧ.

Let us now see how the fascinating plasmonic features of semiconducting GNRs are affected by changes of the in-plane separation. With reference to the case of 5AGNR, we take a positive doping value of 0.2 eV and consider vacuum distances *L*, between continuous arrays, in the range of 5 to 20 Å (Figure 5).

As a first result, we see that both intraband and interband plasmon modes exist in 5AGNR, no matter how far apart the arrays are. The intraband plasmon is, however, affected in intensity, while the interband plasmon is blue-shifted as the vacuum distance decreases down to 5 Å. This effect is clearly visible at *q* = 0.025 Å^−1^ in Figure 5d, where a broad interband plasmon peak is detected at ω ≈ 0.6–1 eV. Both the large blue shift of the interband plasmon, and the intensity decrease of the intraband plasmon, are consistent with the idea that as the GNR arrays get closer a large graphene area is created.

When the vacuum distance becomes negligibly small, the interband plasmon detected in AGNRs enters the region where the π plasmon of graphene are found, while the intraband plasmon decreases in intensity to a small contribution, reported in room temperature calculations of slightly doped graphene [19,31].

**Figure 6:**
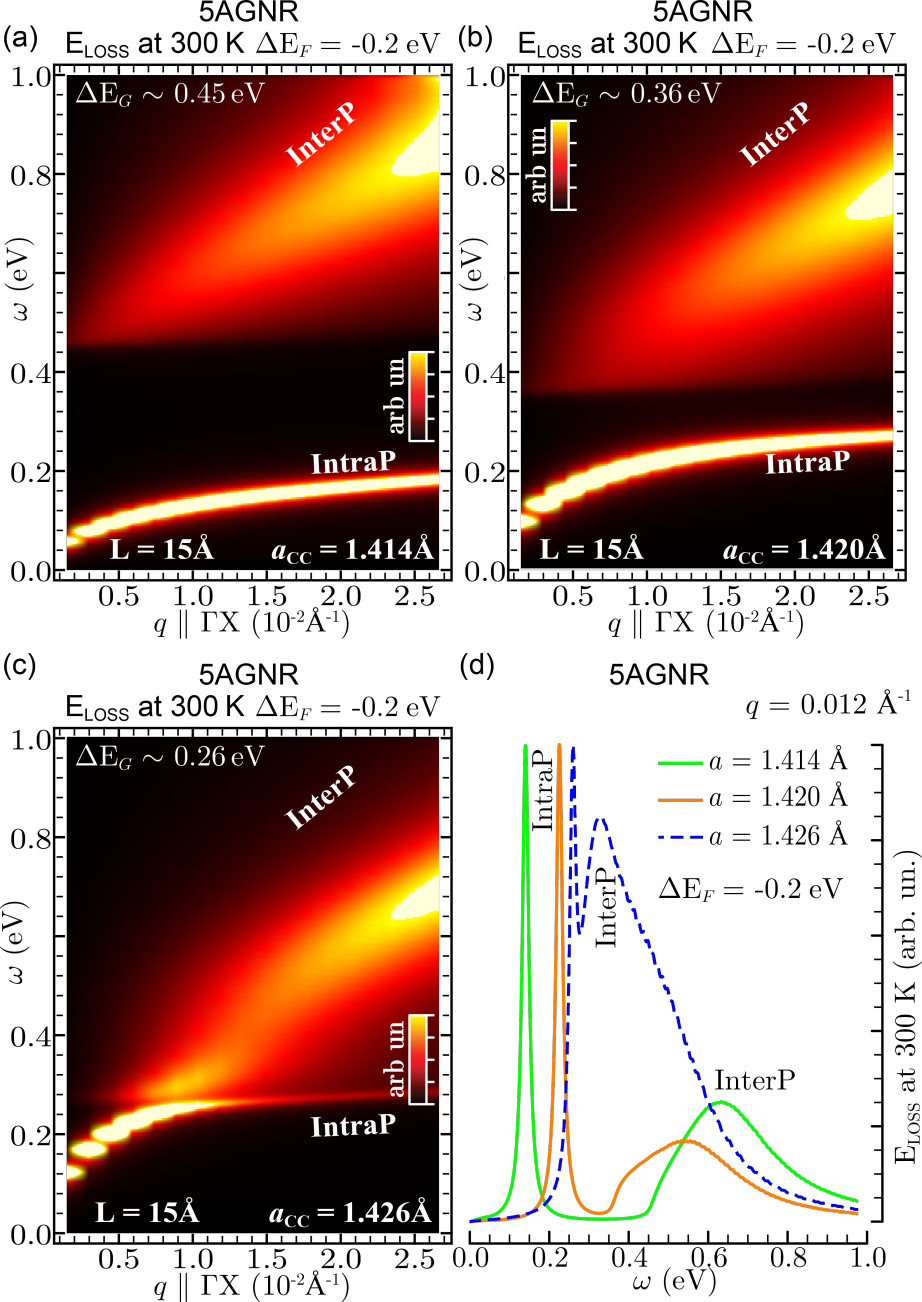
EL function of three negatively doped 5AGNR (Δ*E*_F_ = −0.2 eV), characterized by a C–C bond length *a*_cc_ of 1.411 Å (a), 1.420 Å (b), and 1.426 Å (c), which correspond, respectively, to the band gap values Δ*E*_G_ = 0.45 eV (a), 0.36 eV (b), 0.26 eV (c). In (a–c), the energy–momentum region ω ≤ 1 eV and *q* || ΓX ≤ 0.03 Å^−1^ is explored, showing that *a*_cc_ (or the lattice constant 3*a*_cc_) is a major factor in modulating the intraband and interband plasmons, denoted IntraP and InterP as in Figures 2–5. The intensity scale is cut at 40% of the IntraP peak. The effect of stretching the 5AGNR structure is even more evident in (d), where the different EL functions of (a–c) are compared at a fixed momentum value of *q* = 0.025 Å^−1^ parallel to ΓΧ.

Finally, we show how the intraband and interband plasmons of 5AGNR are affected by stretching/shrinking the unit cell of the system by about 0.5%, with respect to its nominal value associated to a C–C bond length, *a*, of 1.42 Å. In this application, the in-plane vacuum distance is fixed to 15 Å is and a negative doping level of −0.2 eV is considered. As shown in Figure 6, the band gap decreases with increasingly stretching the unit cell from *a* = 1.414 to *a* = 1.426 Å. Accordingly, the interference between the intraband and interband plasmons strongly increases. A similar interference has been reported in undeformed 5AGNR-arrays doped by positive Fermi energy shifts larger than 0.4 eV [31]. However, such doping values seem to be impractical for current GNR applications.

## Conclusion

We have presented a full ab initio modeling, based on ground-state local density calculations, followed by linear response theory, within the RPA, to explore the tunability properties of plasmon excitations in (infinitely periodic) semiconducting (armchair) and semimetallic (zigzag) arrays of GNRs, with ideal symmetric edges, passivated by hydrogen atoms.

All the tested structures are characterized by two interband plasmons at energies larger than 2 eV, which are analogous to the π and π–σ plasmons of graphene. Their peak positions and dispersions are mostly influenced by the GNR width.

At energies smaller than 2 eV, two more intriguing collective excitations appear, which correspond to recently reported edge and surface plasmons [26]. These modes are strongly sensitive not only to the extrinsic conditions, but also to a bunch of geometrical or conformation parameters, such as the width, chirality and unit-cell extension of each GNR, as well as the in-plane vacuum distance between two contiguous GNRs. In ZGNs the absence of a proper LDA band gap, prevents low energy interband excitation from producing a well-defined edge-plasmon structure.

It is worth mentioning that some recent tight-binding models have investigated the role of edge roughness due to asymmetric defects [61–62], which is at present impractical by TDDFT. These studies suggest the edge-plasmon resonances of narrow GNRs are shifted to lower wavelengths and the corresponding plasmon propagation suffers from higher losses with respect to the ideal case, presented here.

We expect that our findings, combined with non-ab initio approaches suitable for the device scale, may open new strategies to construct materials with plasmonic resonances that will be tunable to a specific demand in both the UV–vis and THz regimes, by altering the chemical doping, electronic gating, and also by means of a careful choice of the geometry.

## References

[1] Bao Q, Loh K P (2012). ACS Nano.

[2] Zia R, Schuller J A, Chandran A, Brongersma M L (2006). Mater Today.

[3] Tsargorodska A, Cartron M L, Vasilev C, Kodali G, Mass O A, Baumberg J J, Dutton P L, Hunter C N, Törmä P, Leggett G J (2016). Nano Lett.

[4] Anker J N, Hall W P, Lyandres O, Shah N C, Zhao J, Van Duyne R P (2008). Nat Mater.

[5] Fan W, Lawrie B J, Pooser R C (2015). Phys Rev A.

[6] Haldane F D M (1988). Phys Rev Lett.

[7] Haldane F D M (1991). Phys Rev Lett.

[8] Bernevig B A, Giuliano D, Laughlin R B (2001). Phys Rev Lett.

[9] Bernevig B A, Giuliano D, Laughlin R B (2001). Phys Rev Lett.

[10] Mooij J E, Schön G (1985). Phys Rev Lett.

[11] Giuliano D, Sodano P (2007). Nucl Phys B.

[12] Giuliano D, Sodano P (2010). Nucl Phys B.

[13] Giuliano D, Sindona A, Falcone G, Plastina F, Amico L (2010). New J Phys.

[14] Sindona A, Plastina F, Cupolillo A, Giallombardo C, Falcone G, Papagno L (2007). Surf Sci.

[15] Koppens F H L, Chang D E, García de Abajo F J (2011). Nano Lett.

[16] Castro Neto A H, Guinea F, Peres N M R, Novoselov K S, Geim A K (2009). Rev Mod Phys.

[17] Christensen J, Manjavacas A, Thongrattanasiri S, Koppens F H L, Garcia de Abajo F J (2011). ACS Nano.

[18] Garcia de Abajo F J (2014). ACS Photonics.

[19] Sindona A, Pisarra M, Mencarelli D, Pierantoni L, Bellucci S, Maffucci A, Maksimenko S A (2016). Plasmon Modes in Extrinsic Graphene: Ab initio Simulations vs Semi-classical Models. Fundamental and Applied Nano-Electromagnetics.

[20] Woessner A, Lundeberg M B, Gao Y, Principi A, Alonso-González P, Carrega M, Watanabe K, Taniguchi T, Vignale G, Polini H J (2015). Nat Mater.

[21] Tong J, Muthee M, Chen S-Y, Yngvesson S K, Yan J (2015). Nano Lett.

[22] Sensale-Rodriguez B, Yan R, Kelly M M, Fang T, Tahy K, Hwang W S, Jena D, Liu L, Xing H G (2012). Nat Commun.

[23] Mencarelli D, Bellucci S, Sindona A, Pierantoni L (2015). J Phys D: Appl Phys.

[24] Tao L, Cinquanta E, Chiappe D, Grazianetti C, Fanciulli M, Dubey M, Molle A, Akinwande D (2015). Nat Nanotechnol.

[25] Vacacela Gomez C, Pisarra M, Gravina M, Riccardi P, Sindona A (2016). arXiv.

[26] Fei Z, Goldflam M D, Wu J-S, Dai S, Wagner M, McLeod A S, Liu M K, Post K W, Zhu S, Janssen G C A M (2016). Nano Lett.

[27] Andersen D R, Raza H (2012). Phys Rev B.

[28] Thongrattanasiri S, Manjavacas A, García de Abajo F J (2012). ACS Nano.

[29] Popov V V, Bagaeva T Yu, Otsuji T, Ryzhii V (2010). Phys Rev B.

[30] Wang W, Apell P, Kinaret J (2011). Phys Rev B.

[31] Vacacela Gomez C, Pisarra M, Gravina M, Pitarke J M, Sindona A (2016). Phys Rev Lett.

[32] Gonze X, Amadon B, Anglade P-M, Beuken J-M, Bottin F, Boulanger P, Bruneval F, Caliste D, Caracas R, Côté M (2009). Comput Phys Commun.

[33] Perdew J P, Zunger A (1981). Phys Rev B.

[34] Troullier N, Martins J L (1991). Phys Rev B.

[35] Monkhorst H J, Pack J D (1976). Phys Rev B.

[36] Son Y-W, Cohen M L, Louie S G (2006). Phys Rev Lett.

[37] Yang L, Park C-H, Son Y-W, Cohen M L, Louie S G (2007). Phys Rev Lett.

[38] Raza H, Kan E C (2008). Phys Rev B.

[39] Dubois S M-M, Zanolli Z, Declerck X, Charlier J-C (2009). Eur Phys J B.

[40] Adler S L (1962). Phys Rev.

[41] Wiser N (1963). Phys Rev.

[42] Riccardi P, Sindona A, Barone P, Bonanno A, Oliva A, Baragiola R A (2003). Nucl Instrum Methods Phys Res, Sect B.

[43] Riccardi P, Pisarra M, Cupolillo A, Commisso M, Sindona A, Baragiola R A, Dukes C A (2010). J Phys: Condens Matter.

[44] Petersilka M, Gossmann U J, Gross E K U (1996). Phys Rev Lett.

[45] Onida G, Reining L, Rubio A (2002). Rev Mod Phys.

[46] Despoja V, Dekanić K, Šunjić M, Marušić L (2012). Phys Rev B.

[47] Despoja V, Novko D, Dekanić K, Šunjić M, Marušić L (2013). Phys Rev B.

[48] Novko D, Despoja V, Šunjić M (2015). Phys Rev B.

[49] Pisarra M, Sindona A, Gravina M, Silkin V M, Pitarke J M (2016). Phys Rev B.

[50] Kramberger C, Hambach R, Giorgetti C, Rümmeli M H, Knupfer M, Fink J, Büchner B, Reining L, Einarsson E, Maruyama S (2008). Phys Rev Lett.

[51] Pisarra M, Sindona A, Riccardi P, Silkin V M, Pitarke J M (2014). New J Phys.

[52] Eberlein T, Bangert U, Nair R R, Jones R, Gass M, Bleloch A L, Novoselov K S, Geim A, Briddon P R (2008). Phys Rev B.

[53] Ligato N, Cupolillo A, Sindona A, Riccardi P, Pisarra M, Caputi L S (2014). Surf Sci.

[54] Pisarra M, Riccardi P, Sindona A, Cupolillo A, Ligato N, Giallombardo C, Caputi L (2014). Carbon.

[55] Riccardi P, Cupolillo A, Pisarra M, Sindona A, Caputi L S (2012). Appl Phys Lett.

[56] Pisarra M, Riccardi P, Cupolillo A, Sindona A, Caputi L S (2012). Nanosci Nanotechnol Lett.

[57] Cupolillo A, Pisarra M, Sindona A, Commisso M, Riccardi P (2010). Vacuum.

[58] Generalov A V, Dedkov Yu S (2012). Carbon.

[59] Cupolillo A, Ligato N, Caputi L S (2012). Carbon.

[60] Tao C, Jiao L, Yazyev O V, Chen Y-C, Feng J, Zhang X, Capaz R B, Tour J M, Zettl A, Louie S G (2011). Nat Phys.

[61] Hou H, Teng J, Palacios T, Chua S (2016). Opt Commun.

[62] Li J, Li Z, Zhou G, Liu Z, Wu J, Gu B-L, Ihm J, Duan W (2010). Phys Rev B.

